# Minimal Clinically Important Difference and Patient Acceptable Symptom State After Total Abdominal Hysterectomy: Secondary Analysis of RCT Data

**DOI:** 10.7759/cureus.87703

**Published:** 2025-07-10

**Authors:** Nada Pejcic, Marija Kutlesic, Vladimir Milic, Radmilo Jankovic, Nenad Zornic

**Affiliations:** 1 Clinic for Anesthesiology and Intensive Therapy, University Clinical Center Nis, Nis, SRB; 2 Clinic for Anesthesiology and Intensive Therapy, Clinic for Gynecology and Obstetrics, University Clinical Center Nis, Nis, SRB; 3 Department of Surgery, Faculty of Medical Sciences, University of Kragujevac, Kragujevac, SRB

**Keywords:** mcid, minimal clinically important difference, pass, patient acceptable symptom state, tah, total abdominal hysterectomy

## Abstract

Background

Modern anesthesia focuses on the patient's perception of achieved pain relief rather than just statistical significance. The minimal clinically important difference (MCID) and the patient acceptable symptom state (PASS) are measures of patient-reported outcomes. MCID in pain scores refers to the minimum reduction in visual analogue scale (VAS) or numeric rating scale (NRS) scores that leads to a perceptible improvement in pain, although it may not necessarily indicate full pain relief. PASS, on the other hand, is the threshold beyond which patients consider themselves to be in an acceptable state of well-being. These patient-centered outcomes reflect the clinical significance of the achieved pain relief from the patient's perspective. Furthermore, these metrics can guide anesthesiologists toward postoperative pain management targets. The purpose of this study was to determine the MCID in NRS scores and PASS after total abdominal hysterectomy (TAH).

Methods

This study represents a secondary analysis of a double-blind, randomized controlled trial (RCT) evaluating quadratus lumborum block (QLB) in total abdominal hysterectomy. The primary outcome was to determine the distribution-based MCID in NRS scores. The MCID was calculated as the average of three values derived from distinct distribution-based methods: (1) a proportion of the pooled standard deviation (SD), (2) a proportion of the standard error of measurement (SEM) in the control group, and (3) 10% of the scale range. The secondary outcome was to assess PASS. PASS was determined using the "threshold approach," identifying the pain score threshold that most accurately predicted patient satisfaction with pain relief. This was achieved by determining the value that yielded the best sensitivity and specificity in a receiver operating characteristic (ROC) curve analysis.

Results

The MCID for pain intensity, calculated as the mean value (1.5) derived from three distribution-based methods, represents the threshold for meaningful improvement. Concurrently, an NRS score of ≤3.5 was identified as the PASS threshold, indicating a high probability of patient satisfaction. This value defines the treatment target representing an acceptable level of symptom control.

Conclusion

Our findings establish an MCID of 1.5 and a PASS of ≤3.5 for NRS pain scores following total abdominal hysterectomy, defining clinically meaningful improvement and treatment targets to support more personalized and effective postoperative pain management.

## Introduction

The quadratus lumborum block (QLB) is a regional analgesic technique, a fascial plane block, that involves the injection of a local anesthetic into the quadratus lumborum plane [1]. Lin et al. [2] and Korgvee et al. [3], in their meta-analyses of randomized controlled trials (RCTs), demonstrated a statistically significant reduction in morphine consumption in patients who received posterior QLB as part of multimodal analgesia for various open abdominal procedures (abdominoplasty, inguinal hernia repair, and cesarean section (CS)) and laparoscopic procedures (gynecological surgery, cholecystectomy, colectomy, gastrectomy, and kidney surgery) in the adult population. The European Society of Regional Anaesthesia and Pain Therapy (ESRA) [4] and the Society for Obstetric Anesthesia and Perinatology (SOAP) [5] recommend QLB for post-cesarean pain management in cases where intrathecal morphine cannot be used or for the management of breakthrough pain.

Hysterectomy is the second most common surgical procedure involving the female reproductive system after CS [6]. Although studies have shown a statistically significant reduction in opioid consumption and pain scores following the implementation of QLB in multimodal pain management, modern anesthesia focuses on the patient's perception of achieved pain relief rather than just statistical significance. It is not sufficient to demonstrate mathematical significance; achieving patient satisfaction with pain control and overall well-being is essential. Furthermore, the minimal clinically important difference (MCID) [7] and the patient acceptable symptom state (PASS) are measures of patient-reported outcomes. MCID in pain scores refers to the minimum reduction in visual analogue scale (VAS) or numeric rating scale (NRS) scores that leads to a perceptible improvement in pain, although it may not necessarily indicate full pain relief. PASS, on the other hand, is the threshold beyond which patients consider themselves to be in an acceptable state of well-being [8]. These patient-centered outcomes demonstrate the clinical importance of pain relief achieved from the patient's perspective and can guide anesthesiologists in setting postoperative pain management targets.

Searching the published literature in PubMed, Google Scholar, and the Cochrane Database, we found studies discussing the MCID for chronic pain [9,10], trauma-induced pain [11], and postoperative MCID [12,13]. Some of these studies also addressed the PASS [13]. However, we did not find a recommended MCID for pain intensity or opioid use reduction in patients undergoing total abdominal hysterectomy (TAH).

The purpose of this study was to determine the MCID in NRS scores and PASS after TAH.

## Materials and methods

This study represents a secondary analysis of data from a prospective, double-blind, randomized controlled trial evaluating the analgesic efficacy of the QLB as part of multimodal pain management after TAH, registered on ClinicalTrials.gov (NCT05765318).

The primary outcome of the original QLB trial [14] was cumulative morphine consumption (mg) during the first 12 postoperative hours. Secondary outcomes included pain at rest and during activity (both measured using NRS 0-10), morphine consumption (mg) at 24 hours postoperatively, time to first morphine request, postoperative nausea and vomiting (PONV), sedation, and patient satisfaction with analgesia. Satisfaction was measured on a three-point scale: 0 (dissatisfied), 1 (satisfied), and 2 (very satisfied) with the level of pain relief achieved. To define the PASS, a binary satisfaction variable was created, classifying responses 1 or 2 as "satisfied" and response 0 as "not satisfied".

Pain intensity, PONV, the level of sedation, and satisfaction were assessed at predefined time points: immediately upon arrival in the post-anesthesia care unit (PACU) (T0), two hours thereafter (T2), six hours after arriving in the PACU (T6), 12 hours after arriving in the PACU (T12), and 24 hours after PACU admission (T24), as well as upon every request for additional analgesia.

Surgery was done under general anesthesia. Standardized general anesthesia included induction with a propofol bolus of 1.5-2.5 mg/kg and rocuronium 0.6 mg/kg. Fentanyl 2.5 mcg/kg was given at induction and repeated to keep the blood pressure and heart rate changes up to 20% of baseline. Dexamethasone 0.1 mg/kg was administered IV after the induction of anesthesia as a part of multimodal analgesia. Sevoflurane in a 50% air/50% oxygen mixture with an end-tidal of 2.0 vol% was used as the maintenance agent. Ketoprofen 100 mg IV and metoclopramide 10 mg IV were administered 20 minutes before the end of surgery. All patients received postoperative intravenous ketoprofen 100 mg every 12 hours and intravenous morphine as needed. Morphine was administered by the nurse upon the patient's request if the pain intensity on the NRS was greater than 3/10 (for intensity 4-6/10, morphine 3 mg, and for NRS ≥ 7/10, morphine 5 mg). Pain reevaluation was performed by the nurse 20 minutes after administering morphine. If the pain intensity was still greater than 3, an additional morphine bolus would be given using the same principle, and the pain assessment would be repeated after 20 minutes. The maximum allowed amount of morphine was 40 mg within four hours.

The original study was conducted in accordance with the Declaration of Helsinki and approved by the institutional review board (Ethical Committee of the University Clinical Center Nis, Serbia; approval number: 3007/6, signed on February 2, 2023). Written informed consent was obtained from all patients before inclusion, after explaining all study details, including voluntary participation, data confidentiality, and unrestricted anonymous data usage.

A total of 60 patients were included in the study from March 2023 to February 2024 and were randomly allocated to either the QLB group (bilateral posterior QLB performed at the end of surgery, during anesthesia emergence) or the control group (no block performed). QLB was performed using 30 mL of 0.25% bupivacaine per side. For patients weighing under 60 kg, the total bupivacaine dose was set at 2.5 mg/kg. No QLB-associated complications or adverse effects occurred.

Eligibility criteria included patients scheduled for elective TAH, aged ≥18 years, with a body weight > 50 kg, body mass index (BMI) < 40 kg/m², and an American Society of Anesthesiologists (ASA) physical status I-III. Exclusion criteria included patient refusal to participate in the study, allergies to any study medication, local skin infection at the site of QLB injection, body mass index > 40 kg/m^2^, inability to comprehend or participate in scoring scales, difficult anatomy resulting in poor ultrasound visualization of muscular and fascial structures necessary for correct block administration, and daily regular intake of opioids or any other analgesics.

Outcomes

The primary outcome of this secondary analysis was to determine the distribution-based MCID in NRS scores, which was not addressed in the original study. The secondary outcome was to assess PASS.

Assessment of outcomes

We conducted a distribution-based determination of the MCID for pain intensity. The MCID was calculated as the average of three different values derived from three different distribution-based methods:

${\text{MCID}=\text{average} \left(\text{MCID}_1,\text{MCID}_2,\text{MCID}_3\right)=\frac{1}{3} \times \left(\text{MCID}_1+\text{MCID}_2+\text{MCID}_3\right).}$

MCID_1_ was calculated using the statistical distribution of the NRS scores, presented as the proportion of pooled standard deviation (SD):

{\text{MCID$\unicode{2081}$}=0.5\times \text{pooled} \text{SD}.}

MCID_2_ was determined based on the reliability of the NRS score, as a proportion of the standard error of measurement (SEM) in the control group:

${\text{MCID}_2=1.96 \times \text{SEM}=1.96 \times \text{SDc} \times \sqrt{(1-\text{ICC})},}$

where SDc is the SD of the control group, and ICC is the intraclass correlation coefficient of the control group.

MCID_3_ was defined as 10% of the instrument range.

PASS was determined using the "threshold approach," identifying the pain score threshold that most accurately predicted patient satisfaction with pain relief. This was achieved by determining the value that yielded the best sensitivity and specificity in a receiver operating characteristic (ROC) curve analysis.

Statistics

Data were analyzed using IBM SPSS Statistics version 21.0 (IBM Corp., Armonk, NY). The normality of data distribution was assessed using the Shapiro-Wilk test. Variables were presented as mean (SD), median (interquartile range (IQR)), count (%), or range, as appropriate. Comparisons of normally distributed continuous data were performed using Student's unpaired t-test. The Mann-Whitney U test was used for non-normally distributed data, ranks, and ordinal scores. For binomial data, we applied either the χ² test or Fisher's exact test, as appropriate. The PASS was assessed using ROC curve analysis. All p-values reported are two-sided, with statistical significance set at p < 0.05.

## Results

**Figure 1:**
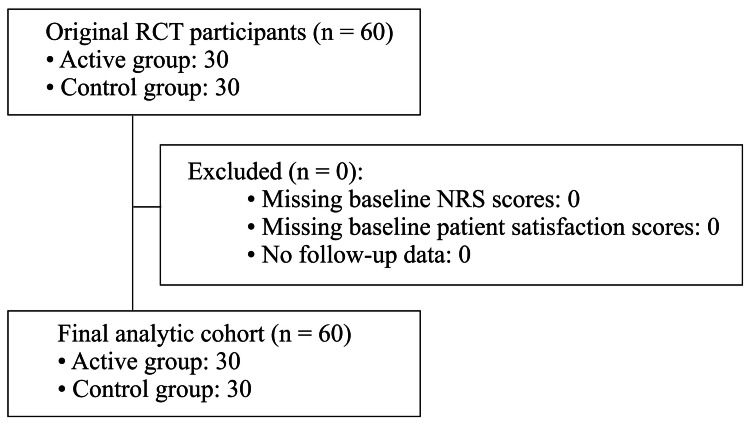
STROBE flow diagram: secondary analysis of RCT data STROBE: Strengthening the Reporting of Observational Studies in Epidemiology, RCT: randomized controlled trial, NRS: numeric rating scale

In the original randomized trial, a total of 60 patients were randomly allocated to two groups (active and control), with 30 participants assigned to each group. NRS and patient satisfaction scores were prospectively collected at five time points with no missing data. Consequently, all 60 patients (30 in the active group and 30 in the control group) were included in the secondary analysis of RCT data (Figure 1).

There were no differences in demographic characteristics, duration of the surgical procedure, or the amount of intraoperatively administered fentanyl between the study groups (Table 1).

**Table 1:** Patient characteristics No statistically significant intergroup difference was observed for any of the variables. ^1^Independent samples t-test ^2^Mann-Whitney U test of independent samples ^3^Fisher's exact test p < 0.05: statistically significant SD: standard deviation, IQR: interquartile range, ASA: American Society of Anesthesiologists, N/A: not applicable

Patients	Control group (n=30)	Active group (n=30)	p-value	Test statistic value
Age, mean (SD)	56.78 (11.13)	56.97 (11.43)	p = 0.950 ^1^	t = -0.063
Body mass (kg), mean (SD)	72.24 (11.26)	72.20 (14.24)	p = 0.991 ^1^	t = 0.012
Body mass index (kg/m^2^), mean (SD)	25.98 (3.30)	26.87 (4.76)	p = 0.420 ^1^	t = -0.813
Surgery duration (minutes), mean (SD)	94.04 (19.24)	102.24 (18.78)	p = 0.116 ^1^	t = -1.599
Fentanyl (mcg/kg/h), median (IQR)	2.28 (2.05, 2.79)	2.49 (1.83, 3.11)	p = 0.949 ^2^	Z = -0.064
ASA physical status, number (%)				
I	4 (13.3)	5 (16.7)	p = 1.0 ^3^	N/A
II	23 (76.7)	22 (73.3)		
III	3 (10.0)	3 (10.0)		

All patients underwent TAH with bilateral salpingo-oophorectomy through a Pfannenstiel incision under general anesthesia.

The MCID in pain intensity was calculated as the average of three values derived from three distribution-based methods (Table 2).

**Table 2:** Patient-centered outcomes after total abdominal hysterectomy MCID: minimal clinically important difference, SD: standard deviation, NRS: numeric rating scale, SEM: standard error of measurement, PASS: patient acceptable symptom state, ROC curve: receiver operating characteristic curve

Patient-centered outcome	Distribution-based method	Value (NRS score)
MCID_1_	Proportion of the pooled SD of NRS scores	0.7605
MCID_2_	Proportion of the SEM of NRS scores in the control group	2.6465
MCID_3_	10% of the measurement range of NRS (0-10)	1
MCID	Average of MCID_1_, MCID_2_,and MCID_3_	1.5
PASS	NRS cutoff for predicting patient satisfaction in a ROC curve	3.5

Proportion of the Pooled SD

\({\text{MCID}_1=0.5\times \text{pooled} \text{SD},}\\ {\text{MCID}_1=0.7605.}\)

SEM Based on the SDc

${\text{MCID}_2=1.96 \times \text{SEM}=1.96 \times \text{SDc} \times \sqrt{(1-\text{ICC})},}$

${\text{MCID}_2 (\text{for}\text{ }\text{ICC}=0.541,\text{SDc}=1.993)=2.6465.}$

10% of the Measurement Range of NRS (0-10)

${\text{MCID}_3=1}$.

Furthermore,

${\text{MCID}=\text{average} \left(\text{MCID}_1,\text{MCID}_2,\text{MCID}_3\right)=\frac{1}{3} \times (0.7605+2.6465+1)=1.469}$,

${\text{MCID}\sim 1.5}$.

We observed a strong negative correlation between QLB performance and pain intensity at rest (Pearson coefficient: -0.679, p < 0.001) as well as pain intensity during activity (Pearson coefficient: -0.658, p < 0.001). None of the patients who received a QLB reported a pain intensity higher than 3/10 on the NRS. Additionally, we found a moderate negative correlation between patient satisfaction and pain intensity at rest (Pearson coefficient: -0.454, p < 0.001) as well as pain intensity during activity (Pearson coefficient: -0.431, p = 0.001). Both correlations were statistically significant at the 0.01 level (two-tailed).

**Figure 2:**
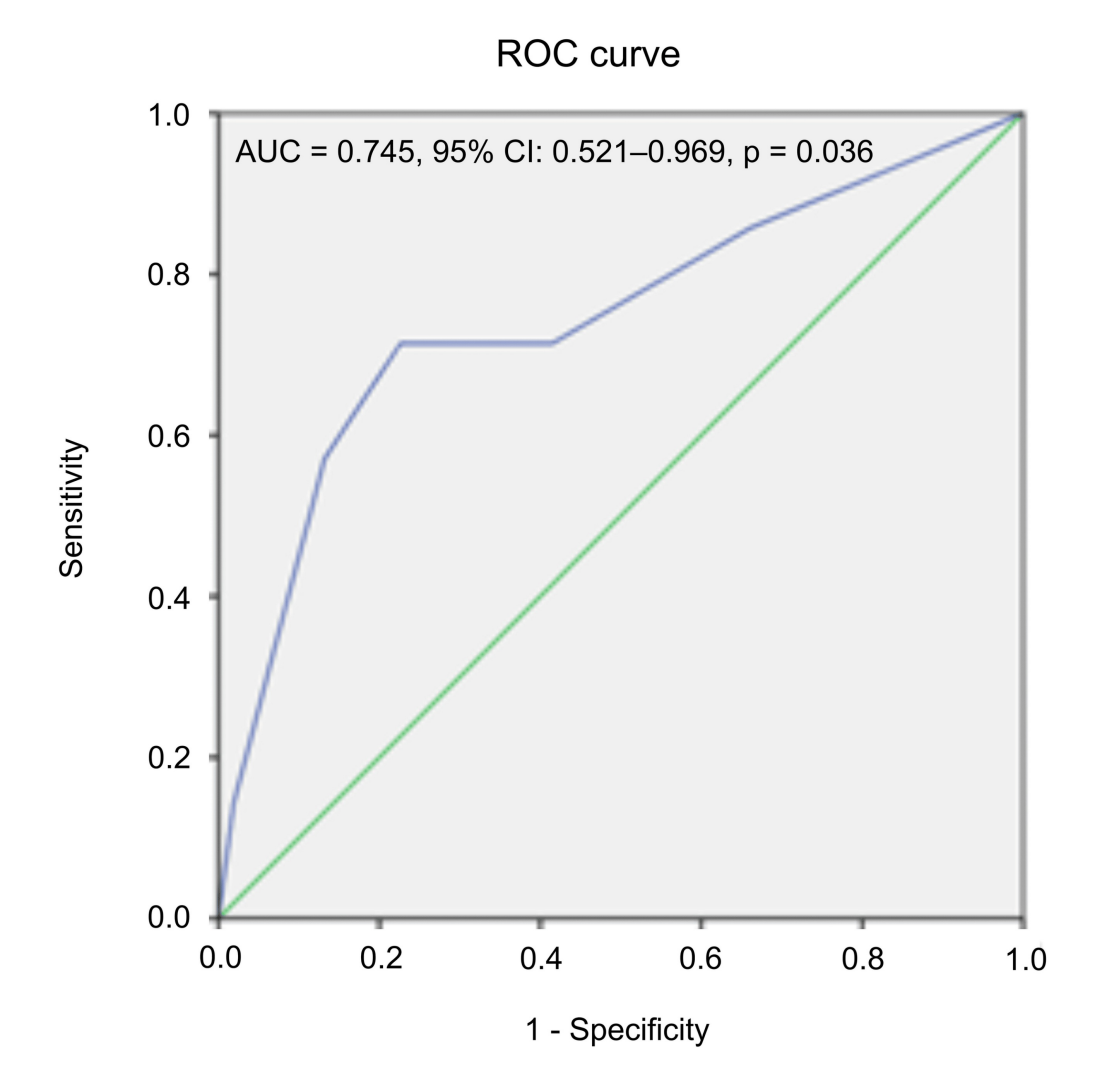
PASS PASS: patient acceptable symptom state, ROC curve: receiver operating characteristic curve, AUC: area under the curve, CI: confidence interval

A ROC curve analysis (Figure 2) was performed to determine the optimal NRS cutoff for predicting patient satisfaction and establishing PASS. The diagonal line (green line) represents a non-discriminatory model (area under the curve (AUC) = 0.5), while the blue line represents the actual model's ability to distinguish between the two satisfaction groups (binary outcome: satisfied versus not satisfied with pain control). The blue line rises above the diagonal (AUC = 0.745, 95% confidence interval (CI): 0.521-0.969, p = 0.036), indicating moderate discriminatory ability. A steeper initial rise suggests that the model effectively identifies true positives early. The optimal NRS cutoff was 3.5, with a sensitivity of 71.4% and a specificity of 77.4%, based on Youden's index (0.488). These findings suggest that an NRS score of 3.5 or lower is associated with a higher probability of patient satisfaction. Therefore, a decrease in the NRS score to 3.5 represents PASS.

Upon arrival at the PACU, all patients who received QLB as part of multimodal pain management achieved PASS (NRS score below the cutoff of 3.5) compared to 13 out of 30 patients (43.33%) in the control group. Two hours later, 25 out of 30 patients (83.33%) in the QLB group had clinically meaningful pain control, compared to 16 out of 30 patients (53.33%) in the control group. Although the original study [14] reported statistically significant differences in NRS scores between the groups in favor of QLB at 12 and 24 hours postoperatively, there was no significant difference in the number of patients who achieved PASS.

## Discussion

Our findings highlight the clinically meaningful pain relief achieved by adding the QLB to multimodal pain management after TAH. We determined that a minimum reduction of 1.5 points in the NRS score was required to produce a perceptible reduction in pain after TAH. Furthermore, to achieve PASS, the pain level needs to decrease to below 3.5 on the NRS.

The MCID is the smallest change in a treatment outcome that a patient would notice. The term MCID was first introduced by Jaeschke et al. [15] in 1989, originally in the context of chronic heart and lung disease improvement. Jaeschke et al. defined MCID as the smallest difference in a score, across any domain or outcome of interest, that patients perceive as beneficial [15]. This concept links the magnitude of change with clinical decision-making and emphasizes the importance of patient perception, which is influenced by various factors such as time, place, and overall health status, leading to significant variability in results.

Although MCID plays a role in pain perception, it is not necessarily sufficient to achieve optimal pain relief. Patients require a greater reduction in pain scores than the MCID threshold to experience satisfactory pain control. One approach to estimating the appropriate level of pain reduction is the PASS. PASS represents the threshold beyond which patients consider themselves well, indicating a symptom level they can tolerate without requiring further treatment [8,9]. Thus, PASS can be used to differentiate responders from non-responders in postoperative pain studies [13]. However, MCID reflects the concept of improvement (feeling better), while PASS represents the concept of overall well-being or symptom remission (feeling good) (Table 3) [16].

**Table 3:** Patient-reported outcomes: MCID and PASS MCID: minimal clinically important difference, PASS: patient acceptable symptom state, VAS: visual analogue scale, NRS: numeric rating scale, SD: standard deviation, SEM: standard error of measurement, ROC curve: receiver operating characteristic curve

Patient-reported outcomes	MCID			PASS
Definition	Minimum reduction in NRS or VAS scores that leads to a perceptible improvement in pain, although it may not necessarily indicate full pain relief			NRS or VAS score threshold that most accurately predicts patient satisfaction with pain relief, beyond which patients consider themselves to be in an acceptable state of well-being and do not require further treatment
	Concept of improvement (feeling better)			Concept of overall well-being or symptom remission (feeling good)
Statistical approach	Distribution-based method (average of MCID_1_, MCID_2_, and MCID_3_)		Anchor-based method	NRS/VAS score cutoff for predicting patient satisfaction by determining the value that yielded the best sensitivity and specificity in an ROC curve
	MCID_1_	Proportion of the pooled SD of NRS/VAS scores	During follow-up visits, patients report their current pain intensity and simultaneously describe how their pain has changed compared to the previous visit using a Likert scale	
	MCID_2_	Proportion of the SEM of NRS/VAS scores in the control group		
	MCID_3_	10% of the measurement range of NRS/VAS		

We observed a 57% reduction in morphine consumption following QLB administration [14]. However, it remains unclear whether this reduction is clinically meaningful. There is no precise definition of how much opioid-sparing, resulting from an analgesic intervention, should be considered clinically significant. Do we only need a reduction in opioid consumption to achieve meaningful pain relief, or is it equally important to minimize opioid-related side effects? What is the relationship between opioid-sparing effects and short- or long-term functional outcomes, quality of recovery, gastrointestinal recovery, mobilization, and sleep quality? Answering these questions requires robust, well-designed studies.

The threshold value determined by the ROC curve suggests that a specific NRS score serves as the best predictor of patient satisfaction with pain relief, balancing sensitivity and specificity. These findings may help identify patients at risk of dissatisfaction and guide improvements in pain management strategies.

The Initiative on Methods, Measurement, and Pain Assessment in Clinical Trials (IMMPACT) reviewed and recommended specific methods for interpreting the clinical significance of treatment outcomes in chronic pain trials [17]. However, there is no consensus in the literature regarding the optimal method for MCID estimation in acute pain.

In our study, we used a distribution-based determination of MCID. Several mathematical models exist for MCID estimation, based on SD and SEM. According to Cohen's effect size, which can be categorized as small, medium, or large, MCID can be calculated as a proportion of SD, using coefficients of 0.2, 0.5, and 0.8, respectively [7]. SD represents the degree of variation within a dataset and can be determined based on the SD of the NRS scores in the control group or by using the pooled SD from both groups.

A more comprehensive estimation of MCID involves comparing changes in patient-reported outcomes (such as NRS or VAS scores) to a patient-reported "anchor." During follow-up visits, patients report their current pain intensity and simultaneously describe how their pain has changed compared to the previous visit, using a Likert scale (e.g., minimal or significant improvement/worsening). The major advantage of the anchor-based approach is that it directly links pain score estimation to patients' self-reported experience. However, it also has significant limitations. This method is strongly influenced by the patient's current state but only weakly reflects their previous state. Additionally, patients may not accurately recall their baseline health status, particularly in the immediate postoperative setting, where sedative and opioid medications can affect memory and perception [18].

Although VAS and NRS are incomplete representations of the multidimensional aspects of pain and cannot fully capture the pain experience, they remain the most widely used metrics for assessing postoperative pain. Pain intensity reduction measured by NRS/VAS provides a crucial link between analgesic consumption (typically opioids) and the patient's perception of clinical improvement resulting from a specific treatment [12]. MCID and PASS can help identify a suitable target for optimal analgesic titration. Furthermore, MCID can be used to guide the non-inferiority margin in clinical trials, to determine the sample size for a clinical trial, to define the threshold for clinical relevance in positive findings, and to establish the minimum detectable difference for negative findings [19].

A patient's perception of pain improvement or deterioration can be influenced by biological, psychological, and social factors to varying degrees [12]. First, it is essential to understand the patient's expectations, just as we consider patient satisfaction [20]. The experience of pain improvement is non-linear. Acute pain is typically more responsive to treatment than chronic pain. Additionally, the intensity of pain at the moment of treatment strongly influences the perceived effectiveness of pain relief: the higher the initial pain level, the more potent the required analgesic treatment [21]. Pain intensity and the perception of pain relief can also be influenced by age, cultural background, and ethical norms. A key question is whether the MCID for acute pain depends on the type of procedure or the procedure-specific pain intensity. Is the MCID for postoperative pain relief the same for all patients, or does it vary based on the presence of chronic preoperative pain?

Previous studies suggest that reductions in pain scores of approximately 30%-40% are necessary to reflect clinically meaningful pain improvement [13]. Olsen et al. reported a wide range of absolute MCID values (ranging from 8 to 40 on a 100 mm scale) and relative MCID values (ranging from 13% to 85%), concluding that MCID is influenced by both the definition of improvement and the study design [21].

Patients presenting to emergency departments with various pain conditions reported an MCID for the VAS score ranging from 12 to 18 [11], compared to patients with chronic pain, who estimated MCID as 2 of 10 in the NRS (comparable to VAS 20 of 100 mm) [9,10]. Myles et al. determined MCID and PASS for the VAS score in patients with acute pain after CS, reporting a MCID of 9.9 mm (rounded to 10 mm) and a PASS of 33 mm [13]. These findings are comparable to our results, where MCID and PASS for the NRS score were 1.5 and 3.5, respectively.

Several publications have addressed MCID for pain in rheumatologic, orthopedic, and degenerative spine diseases requiring surgery [22-24]. However, these MCID values are not directly comparable to those for pain intensity following CS or TAH due to differences in pain characteristics and preoperative duration of pain.

While acknowledging its limitations, this study delivers new insights into postoperative pain management after TAH by quantifying patient-centered outcomes not previously reported.

Our study has several limitations. First, we had a sample size of 60 patients in a single center, which raises concerns about the generalizability to other populations or healthcare settings. We provided a distribution-based calculation of MCID. The lack of anchor-based MCID validation and reliance on distribution-based methods alone slightly weaken generalizability. Future studies with larger sample sizes and different study designs, including both distribution-based and anchor-based MCID determination, are needed to correlate distribution-based MCID with patient-reported anchor-based assessments.

## Conclusions

The QLB significantly improves immediate postoperative pain control and patient satisfaction after TAH compared to standard analgesia. QLB should be integrated into multimodal analgesia protocols for TAH to ensure rapid, meaningful pain relief and high patient satisfaction in the critical early recovery phase. Our findings also establish the MCID and PASS thresholds for NRS pain scores following TAH. These novel, patient-derived values bridge research and clinical practice by defining clinically meaningful improvement and treatment success, enabling more personalized and effective postoperative pain management.
